# Phytochemical and Biological Investigation of an Indigenous Plant of Bangladesh, *Gynura procumbens* (Lour.) Merr.: Drug Discovery from Nature

**DOI:** 10.3390/molecules28104186

**Published:** 2023-05-19

**Authors:** Md. Abu Jobaer, Sania Ashrafi, Monira Ahsan, Choudhury Mahmood Hasan, Mohammad Abdur Rashid, Sheikh Nazrul Islam, Mohammad Mehedi Masud

**Affiliations:** 1 Phytochemical Research Laboratory, Department of Pharmaceutical Chemistry, Faculty of Pharmacy, University of Dhaka, Dhaka 1000, Bangladesh; abu.jobaer60@gmail.com (M.A.J.); moniraahsan@du.ac.bd (M.A.); cmhasan@gmail.com (C.M.H.); rashidma@du.ac.bd (M.A.R.); 2 Institute of Nutrition and Food Science, University of Dhaka, Dhaka 1000, Bangladesh; sheikhnazrul09@gmail.com

**Keywords:** *Gynura procumbens*, phytol, lupeol, stigmasterol, β-sitosterol, friedelanol acetate, β-amyrin, NMR, anti-diabetic activity

## Abstract

*Gynura procumbens* (Lour.) Merr. (Family: Asteraceae) is a tropical Asian medicinal plant found in Thailand, China, Malaysia, Indonesia, and Vietnam. It has long been utilized to treat a variety of health concerns in numerous countries around the world, such as renal discomfort, constipation, diabetes mellitus, rheumatism, and hypertension. The chemical investigation resulted in the isolation and characterization of six compounds from the methanol (MeOH) extract of the leaves of *Gynura procumbens*, which were identified as phytol (**1**), lupeol (**2**), stigmasterol (**3**), friedelanol acetate (**4**), β-amyrin (**5**), and a mixture of stigmasterol and β-sitosterol (**6**). In-depth investigations of the high-resolution ^1^H NMR and ^13^C NMR spectroscopic data from the isolated compounds, along with comparisons to previously published data, were used to clarify their structures. Among these, the occurrence of Compounds **1** and **4** in this plant are reported for the first time. The crude methanolic extract (CME) and its different partitionates, i.e., petroleum ether (PESF), chloroform (CSF), ethyl acetate (EASF), and aqueous (AQSF) soluble fractions, were subjected to antioxidant, cytotoxic, thrombolytic, and anti-diabetic activities. In a DPPH free radical scavenging assay, EASF showed the maximum activity, with an IC_50_ value of 10.78 µg/mL. On the other hand, CSF displayed the highest cytotoxic effect with an LC_50_ value of 1.94 µg/mL compared to 0.464 µg/mL for vincristine sulphate. In a thrombolytic assay, the crude methanolic extract exhibited the highest activity (63.77%) compared to standard streptokinase (70.78%). During the assay for anti-diabetic activity, the PESF showed 70.37% of glucose-lowering activity, where standard glibenclamide showed 63.24% of glucose-reducing activity.

## 1. Introduction

Plants have created a variety of chemicals to defend themselves from animal attacks and environmental damage due to the numerous stresses and difficulties they face along with their sedentary lifestyle [1]. In the past, numerous plants were noted as having therapeutic characteristics and were utilized to cure a variety of pathological disorders [2,3,4,5,6]. In particular, for anticancer and antimicrobial medicines, natural compounds from biodiversity have been the preferred repository of medications [7,8,9,10]. Although contemporary therapy has supplanted conventional medicine as a technique for treating human illness [11,12,13], the application of herbal remedies for illness prevention and treatment has expanded during the past few decades in many countries, including developed nations [14,15,16,17,18,19]. Many therapeutic plant extracts are currently utilized as prescription medications in a number of developed countries, including the USA, the UK, Germany, China, and France. For example, herbal supplements containing *Eleutherococcus senticosus* and *Rhodiola rosea* are widely available in the USA and Europe for their numerous pharmacological activities [20,21]. Natural products can act as innovative prototypes for future drug development and structural changes due to their biological and molecular variety, leading to the development of more effective and secure medicines. Thanks to advancements in technology, natural products are scrutinized and evaluated more effectively than ever. As a result, natural compounds are expected to remain the best suppliers of unique, profitable drug lead candidates [7].

*Gynura procumbens* (Lour.) Merr. (commonly referred to as Sambung Nyawa or Sabungai) belongs to the family of Asteraceae, sometimes called “longevity spinach”, which grows extensively in Southeast Asia, mainly in Malaysia, Indonesia, and Thailand [22,23]. This plant is reportedly prevalent in Bangladesh and is locally known as “diabetes leaf”. It has a fleshy stem, a height range of 1–3 m, and a purple hue. The leaves are lanceolate or ovate-elliptic, measuring 0.8 to 3.5 cm in width and 3.5 to 8 cm in length. Flower heads are panicled, slender, yellow, and between 1 and 1.5 cm long [24].

The plant is well known for its diverse phytochemicals and pharmacological properties. It has a long history of use as a herbal remedy, particularly in Southeast Asia. Numerous conditions are treated with the plant, including cancer, diabetes mellitus, eruptive fever, kidney illness, rash, inflammation, and hypertension [25,26,27]. In Malaysia, *G. procumbens* leaves are frequently consumed raw, whereas in Thailand, the leaves are also cooked [28]. *G. procumbens* is often referred to as Sambung Nyawa in Malay, meaning “prolongation of life”, and Bai Bing Cao in Mandarin, meaning “100 illnesses” [29]. This is because it is applied topically and systemically in traditional medicine to treat a wide range of illnesses [30]. For instance, it is frequently used in Indonesia to ease kidney discomfort, and in Vietnam, it is used to treat fever. It is extensively used in Thailand to treat inflammation, rheumatism, and viral diseases [29]. In rats with diabetes produced by streptozotocin (STZ), the methanol and ethanol extracts made from the plant’s leaves were reported to have an anti-diabetic effect [31,32]. The leaves of the plant are frequently ingested in food, and research has demonstrated that the contents of the leaves are not poisonous [33]. A multitude of studies have revealed the presence of various active chemical components, including saponins, flavonoids, terpenoids, tannins, and sterol class of glycosides, in different extracts of *G. procubens* leaves [34,35]. Rutin, kaempferol, and two possible antioxidant substances—kaempferol-3-*O*-rutinoside and astragalin—have also been found in *G. procubens* leaf extract, according to earlier investigations [36]. With this species, only one study project has been carried out in Bangladesh. Thus, the plant was subjected to phytochemical and pharmacological studies, and in this study, we report the separation and identification of six compounds from the ethyl acetate (EtOAc)-soluble fraction of the methanol crude extract of the *G. procumbens* leaves growing in Bangladesh. We also report the antioxidant, cytotoxic, thrombolytic, and antidiabetic activities of the leaves of *G. procumbens.* The animal model is used to deal with animals other than humans as it may mimic disease progression, diagnosis, and treatment similar to in humans. The discovery of a drug and/or component, equipment, toxicological tests, dosing, and side effects are all examined in vivo for future use in humans, while ethical problems are taken into account. Herein lies the significance of the animal model in biomedical research. Based on the literature review, ethnomedicinal usage of the plant, and the chemical nature of the isolated compounds, biological tests were chosen.

## 2. Results and Discussion

### 2.1. Isolated Phytochemicals from G. procumbens

**Figure 1:**
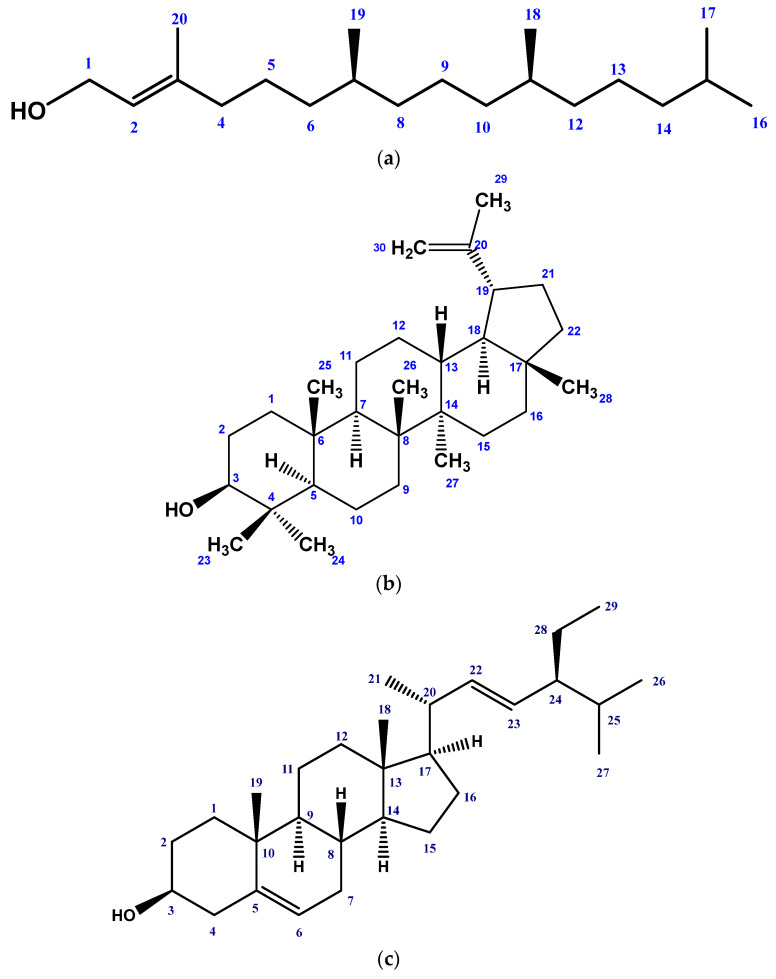

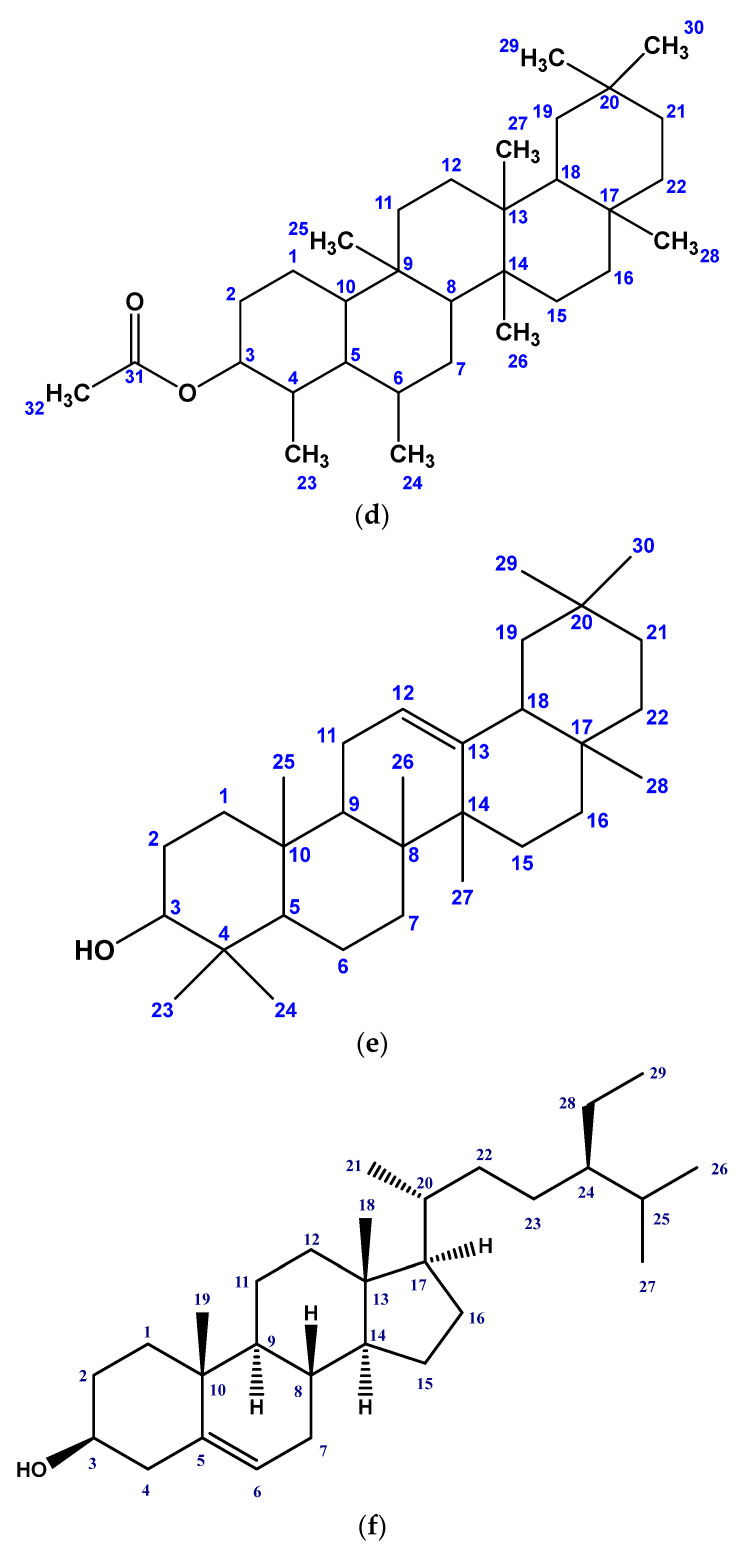
Structures of isolated phytochemicals from *Gynura procumbens* using NMR technologies: (**a**) Phytol, (**b**) Lupeol, (**c**) Stigmasterol, (**d**) Friedelanol acetate, (**e**) β-amyrin, and (**f**) β-sitosterol.

Six compounds were obtained from the EtOAc-soluble portion of the MeOH crude extract of *G. procumbens* by repeatedly using chromatography and purifying on top of the silica gel. The structures of the isolated compounds were identified as phytol (**1**), lupeol (**2**), stigmasterol (**3**), friedelanol acetate (**4**), β-amyrin (**5**), and a mixture of stigmasterol and β-sitosterol (**6**) (Figure 1) by meticulously analyzing their high-quality ^1^H and ^13^C NMR spectroscopic data, and comparing with the published values. 

Phytol ((2E,7R,11R)-3,7,11,15-tetramethyl-2-hexadecen-1-ol) (**1**): Greenish yellow oily liquid, soluble in ethyl acetate and chloroform; ^1^H NMR (400 MHz, CDCl_3_): δ4.17 (2H d, *J* = 7.8 Hz, H-1), 5.43 (1H br t, *J* = 6.8 Hz, H-2), 1.98 (2H t, *J* = 7.0 Hz, H-4), 1.07 (1H m, H-7), 1.07 (1H m, H-11), 1.54 (1H m, H-15), 0.87 (6H d, *J* = 6.8 Hz, H-16,17), 0.86 (6H d, *J* = 6.8 Hz, H-18,19), 1.66 (3H s, H-20). ^13^C NMR (100 MHz, CDCl_3_): δ59.43 (C-1), 123.13 (C-2), 140.30 (C-3), 39.87 (C-4), 25.15 (C-5), 36.67 (C-6), 32.70 (C-7), 37.30 (C-8), 24.47 (C-9), 37.43 (C-10), 32.79 (C-11), 37.37 (C-12), 24.78 (C-13), 39.38 (C-14), 27.97 (C-15), 22.60 (C-16), 22.69 (C-17), 19.71 (C-18), 19.74 (C-19), 16.16 (C-20).

Lupeol (**2**): White amorphous solid, soluble in ethyl acetate and chloroform; ^1^H NMR (400 MHz, CDCl_3_): δ3.20 (dd, *J* = 4.5 Hz, 11.0 Hz, H-3), 0.77 ((m, H-5), 1.41 (1H, m); 1.38 (1H, m), H-6), 2.33 (dt, *J* = 5.5 Hz, 11.0 Hz, H-19), 1.92 (1H, m); 1.27 (1H, m), H-21, 0.93 (s, H-23), 0.70 (s, H-24), 0.88 (s, H-25), 1.02 (s, H-26), 0.97 (s, H-27), 0.81 (s, H-28), 4.69 (d, *J* = 2.5 Hz; 4.59 s, H-29), 1.66 (s, H-30).

Stigmasterol (**3**): White crystal, soluble in ethyl acetate and chloroform; ^1^H NMR (400 MHz, CDCl_3_): δ 3.52 (1H m, H-3), 5.34 (1H d, *J* = 8.2 Hz, H-6), 0.70 (3H s, H-18), 1.03 (3H s, H-19), 0.93 (3H d, *J* = 6.5 Hz, H-21), 5.15 (1H dd, *J* = 15.2, 8.0 Hz, H-22), 5.03 (1H dd, *J* = 15.2, 8.0 Hz, H-23), 0.84 (3H d, *J* = 6.5 Hz, H-26), 0.81 (3H t, *J* = 6.5 Hz, H-29) 

Friedelanol acetate (**4**): White crystal, soluble in ethyl acetate and chloroform; ^1^H NMR (400 MHz, CDCl_3_):δ 4.52 (1H, m, H-3), 1.85 (1H, dt, *J* = 10.4, 2.4 Hz, Ha-2), 1.73 (1H, dt, *J* = 12.8, 3.2 Hz, Ha-6), 0.91 (3H, d, *J* = 6.8 Hz, H_3_-23), 0.97 (3H, s, H_3_-24), 0.82 (3H, s H_3_-25), 0.99 (3H, s, H_3_-26), 1.01 (3H, s, H_3_-27), 1.11 (3H, s, H_3_-28), 0.90 (3H, s, H_3_-29), 1.0 (3H, s, H_3_-30), 2.07 (3H, s, H_3_-32).

β-amyrin (**5**): Amorphous powder, soluble in ethyl acetate and chloroform; ^1^H NMR (400 MHz, CDCl_3_): δ 3.20 (dd, *J* = 10.5 and 4.7 Hz, H-3), 5.16 (m, H-12), 0.92 (s, H_3_-23), 0.90 (3H, s, H_3_-24), 0.99 (3H, s, H_3_-25), 0.97 (3H, s, H_3_-26), 1.08 (3H, s, H_3_-27), 0.85 (3H, s, H_3_-28), 0.88 (3H, s, H_3_-29), 0.89 (3H, s, H_3_-30).

Mixture of Stigmasterol and β-Sitosterol (**6**): Needle-shaped crystal, soluble in ethyl acetate and chloroform; Stigmasterol: ^1^H NMR (400 MHz, CDCl_3_):δ 3.51 (m, H-3), 5.343 (br.s, H-6), 0.688 (3H, s, Me-18), 1.0 (3H, s, Me-19), 1.02 (d, *J* = 7.5 Hz, H-21), 5.01 (1H, m, H-22), 5.14 (1H, m, H-23), 0.79 (d, *J* = 6.5 Hz, H-26), 0.85 (d, *J* = 6.5 Hz, H-27), 0.81 (t, *J* = 7.5 Hz, H-29). β-Sitosterol: ^1^H NMR (400 MHz, CDCl_3_): δ 3.51 (m, H-3), 5.343 (br.s, H-6), 0.68 (3H, s, Me-18), 1.02 (3H, s, Me-19), 0.91 (d, *J* = 6.4 Hz, H-21), 0.809 (d, *J* = 6.4 Hz, H-26), 0.82 (d, 3H *J* = 6.4 Hz, H-27), 0.85 (t, *J* = 7.4 Hz, H-29).

The ^1^H NMR spectrum (400 MHz, CDCl_3_) of Compound **1** showed one doublet of two-proton intensity at δ 4.17 (*J* = 6.8 Hz), which could be assigned to H-1. A broad triplet of one-proton intensity viewed at δ 5.43 was attributed to the olefinic methine (=CH-). The triplet at δ 1.98 of two-proton intensity was attributed to H-4. A multiplet at δ 1.07 at C-2 indicated the occurrence of two protons at H-7 and H-11. The methyl protons (H-20) attached to the C-3 were detected as a broad singlet methyl group proton at δ 1.69. A doublet at δ 0.86 for six protons was allocated to the locations at C-7 and C-11. Again, another doublet at δ 0.87 (*J* = 6.8 Hz) was assigned to six methyl protons (H_3_-16, H_3_-17) attached to C-15. The ^13^C NMR spectrum (100 MHz, CDCl_3_) of Compound **1** exhibited signals for 20 carbons with δ values characteristic of diterpene alcohol phytol. The ^1^Hand ^13^CNMR data were compared to those of phytol [37] and were found to be identical. Therefore, Compound **1** was distinguished as phytol. This is the first report of its isolation from *G. procumbens* (Supplementary Materials: Figures S1–S3).

The ^1^H NMR (400 MHz, CDCl_3_) spectrum of Compound **2** displayed a double doublet (*J* = 11.0, 4.5 Hz) of one-proton intensity at δ 3.20, which could be ascribed to H-3 in a triterpene skeleton. The occurrence of three proton singlets at δ 0.70, 0.81, 0.88, 0.97, 0.93, and 1.02 were attributed to the methyl protons at H-24, H-28, H-25, H-27, H-23, and H-26, respectively. The multiplet of one-proton intensity at δ 2.33 was ascribed to H-19. An isopropenyl side chain with signals at δ 1.66 (3H, s, H-30) and two broad signals at δ 4.69 and 4.59 (each 1H, s, H-29a and H-29b) demonstrated a lupane-type triterpenoid skeleton. The aforementioned spectral characteristics were comparable to those documented for lupeol. Based on this, Compound **2** was identified as lupeol [38] (Figure S4).

The ^1^H NMR spectrum (400 MHz, CDCl_3_) of Compound **3** showed a one-proton multiplet at δ 3.52, the position and multiplicity of which was characteristic of the H-3 of the steroidal nucleus. The characteristic signal for the olefinic H-6 of the steroidal skeleton was obvious from a doublet at δ 5.34 (*J* = 8.2 Hz) that revealed integration for one proton. The trans-olefinic protons (H-22 and H-23) were exhibited as characteristic downfield signals at 5.15 (dd, *J* = 15.2, 8.0 Hz) and 5.03 (dd, *J* = 15.2, 8.0 Hz), respectively, in the ^1^H NMR spectrum due to couplings with the neighboring olefinic and methine protons. The spectrum further revealed signals at δ 0.70 and 1.03 (3H each), which were assigned to two tertiary methyl groups at C-13 and C-10, respectively. The ^1^H NMR spectrum also showed a doublet of three-proton intensity centered at δ 0.91 (d, *J* = 6.4 Hz) and a triplet (3H, *J* = 6.5 Hz) at δ 0.81 for the methyl groups at C-20 and C-28, respectively. The above spectral features are in close agreement with those observed for stigmasterol [39]. Thus, the identity of Compound **3** was characterized as stigmasterol (Figure S5).

The ^1^H NMR spectrum (400 MHz, CDCl_3_) of Compound **4** displayed a proton resonance at δ 4.52 (1H, m), which could be allocated to the oxymethine proton at C-3. However, its downfield resonances implied that C-3 was esterified. This was further supported by a three-proton singlet at 2.07 (3H, s), which could be attributed to the CH_3_CO group. The ^1^H NMR spectrum of Compound **4** exhibited a pair of doublets of triplets centered at δ 1.85 (1H, dt, *J* = 10.4, 2.4 Hz) and 1.73 (1H, dt, *J* = 12.8, 3.2 Hz), which could be assigned to Ha-2 and Ha-6, respectively. The chemical shift and splitting of this proton signal were typical of the friedelanol-type triterpenoid skeleton. The ^1^H NMR spectrum also confirmed one three-proton doublet at δ 0.91 (H_3_-23) and seven three-proton singlets at δ 0.97 (H_3_-24), 0.82 (H_3_-25), 0.99 (H_3_-26), 1.01 (H_3_-27), 1.11 (H_3_-28), 0.91 (H_3_-29), and 1.10 (H_3_-30). The aforementioned spectral characteristics were comparable to those reported for friedelanol acetate [40]. On the basis of the spectral data, Compound **4** was characterized as friedelanol acetate. This is the first report of its isolation from *G. procumbens* (Figure S6).

The ^1^H NMR spectrum (400 MHz, CDCl_3_) of Compound **5** demonstrated the occurrence of eight methyl (3H) singlets at δ 0.58 (H_3_-28), 0.88 (H_3_-29), 0.89 (H_3_-30), 0.90 (H_3_-24), 0.92 (H_3_-23), 0.99 (H_3_-25), 0.97 (H_3_-26), and 1.08 (H_3_-27), and a broad singlet centered at δ 5.16 (H-12) typical for an oleanane-type carbon skeleton. The double doublet (*J* = 10.5 and 4.7 Hz) centered at δ 3.20 can be demonstrated for a β-oriented proton at C-3. The mentioned spectral features were substantially in accord with those observed for β-amyrin [41], and Compound **5** was characterized as β-amyrin (Figure S7).

The ^1^H NMR (400 MHz, CDCl_3_) data were compared to those of stigmasterol and β-sitosterol [39] and were found to be similar. Thus, the identification of Compound **6** was confirmed as a mixture of stigmasterol and β-sitosterol (Figure S8).

### 2.2. Effect of G. procumbens Extracts on DPPH Free Radical Scavenging Activity

**Figure 2:**
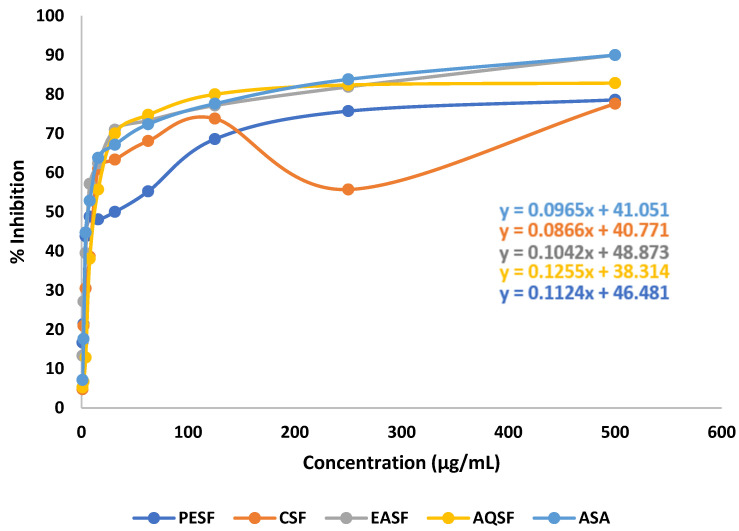
Linear regression equations (IC_50_) of ascorbic acid (ASA) and various extracts of *Gynura procumbens*.

Various extracts of *G. procumbens* demonstrated dose-dependent free radical scavenging activity in contrast to ascorbic acid (standard) in the DPPH free radical scavenging research. In this study, the ethyl acetate soluble fraction of the leaves of *G. procumbens* (EASF) showed significant free radical scavenging activity with an IC_50_ value of 10.78 µg/mL, while the standard ascorbic acid (ASA) showed an IC_50_ value of 31.25 µg/mL (Table 1). The linear regression equation was used to attain the IC_50_ values for ASA and the fractions, and the results are shown in Figure 2.

### 2.3. Effect of G. procumbens Extracts on Brine Shrimp Lethality Bioassay

**Figure 3:**
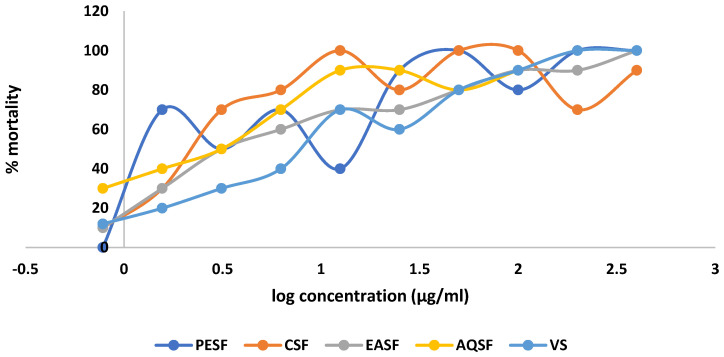
Linear regression equations (LC_50_) of vincristine sulphate (VS) and various extracts of *Gynura procumbens*.

In the brine shrimp lethality bioassay, the chloroform-soluble fraction of the leaves of *G. procumbens* (CSF) displayed the highest cytotoxic effect with an LC_50_ value of 1.94 µg/mL compared to 0.464 µg/mL for vincristine sulphate (VS), whereas the aqueous soluble fraction (AQSF) also showed significant effects with LC_50_ values of 2.07 µg/mL (Table 1). The linear regression analysis is shown in Figure 3. 

### 2.4. Effect of G. procumbens Extracts on the Thrombolytic Activity

In the thrombolytic activity study, the crude methanolic extract showed 63.77% clot lysis, whereas the standard streptokinase showed 70.78% clot lysis (Table 1). This supported the strong thrombolytic activity of the crude extract. Among the extracts, petroleum-ether-soluble fraction (PESF), CSF, and EASF presented moderate thrombolytic activity compared to the standard.

### 2.5. Effect of G. procumbens Extracts on the Anti-Diabetic Activity

The methanolic extract of the leaves of *G. procumbens* and its various fractions exhibited statistically significant (*p* < 0.001) blood-glucose-lowering activity at a dose of 250 mg/kg body weight. The repeated oral administration of CME, PESF, CSF, EASF, and AQSF fractions of *G. procumbens* into diabetic rats for different days caused significant reductions in the blood glucose levels, of 52.82%, 70.37%, 62.72%, 53.85%, and 60.99%, respectively, whereas glibenclamide (standard) reduced the blood glucose by 63.24%. The PESF, CSF, and AQSF fractions showed greater glucose-reducing activity than the other fractions. PESF revealed the highest glucose-lowering activity of 70.37% (5.78 mmol/L) compared to the standard glibenclamide, which showed 63.24% (5.16 mmol/L) glucose-lowering activity. (Table 2 and Table 3).

### 2.6. Analysis of the Biological Investigation 

In previous centuries, natural products have played a significant role in the development of new medications, in particular for the treatment of infectious and cancerous diseases, but also for the treatment of other therapeutic conditions, such as cardiovascular disease (for example, statins) as well as multiple sclerosis (for example, fingolimod) [42]. Significant scaffold diversity and structural complexity define natural products. In comparison to synthetic compound libraries, they characteristically have a greater number of sp3 oxygen and carbon atoms, lesser nitrogen and halogen atoms, larger molecular masses, smaller calculated octanol–water partition coefficients (cLogP values, indicating higher hydrophilicity), additional H-bond acceptors and donors, and better molecular rigidity [43,44,45,46,47]. These characteristics may be favorable; for instance, the higher rigidity of natural products may be favorable in the drug development to address protein–protein interactions [48]. The phytochemicals that are extracted from various plant extracts are typically connected to their biological activities.

Antioxidants are molecules that are capable of preventing reactive oxygen species (ROS) from being produced [49]. They are necessary for the defense against oxidative stress and the negative consequences of ROS inside the body [50]. Phenolic acids, flavonoids, and terpenoids are a few examples of well-known antioxidants [51]. Numerous research has shown that acyclic diterpene phytol (**1**) is an effective cytotoxic, antibacterial, anticonvulsant, antioxidant, anti-inflammatory, antinociceptive, and immune-modulating substance [52,53,54,55,56]. The chemical structure of phytol may have a substantial role in the antioxidant activities of the plant shown in the study. The hydrogen in the alcohol group can scavenge free radicals, while the double bond present in phytol can aid in the formation of the stable free radical’s resonance structure. Although saturated aliphatic alcohols do not generally have high antioxidant properties, phytol does because of the alcohol group’s allylic nature. As a matter of fact, the double bond may play a role in the production of an intermediate resonance structure for the hypothesized mechanism, where the oxygen can create a double bond with the neighboring carbon and the double bond switches to 3,4 carbons [56]. Lupeol (**2**) is a pentacyclic triterpene that exhibits antioxidative activity through the direct scavenging of free radicals and protects the membrane permeability [51]. β-Amyrin (**4**) has a phenolic hydroxyl group and has the capacity to receive electrons. These electrons can compete with free radicals to lessen the lipid peroxidation caused by free radicals [57]. More study is necessary to fully comprehend the core antioxidant mechanism of our distinct phytochemicals. 

Recent studies have shown that phytol (**1**) has the ability to kill specific cancer cell lines, including those from breast, lung, cervical, melanoma, colorectal, and prostate adenocarcinoma [54], most likely by inducing apoptosis [58]. Phytosterols might show anticancer effects through cell cycle arrest and apoptosis [59]. Apoptosis, inhibiting the migration and invasion of cancer cells, and suppressing cell proliferation are molecular mechanisms associated with the anticancer activities of Lupeol (**2**) and β-amyrin (**4**) [60,61]. Further investigation is required to fully understand the fundamental cytotoxicity activities of our separated phytochemicals. 

Diabetes mellitus (DM) is a complex condition defined by elevated blood sugar levels brought on by deficiencies in insulin secretion and/or action. Diabetes is typically treated by altering either the lipid or glucose metabolism, or both [62,63]. Developing diabetes complications is a global health issue that millions of people must deal with. Nearly 10% of people worldwide are affected by this serious endocrine condition [64]. Globally, the number of diabetics has been rising quickly; by 2030, there will be 439 million cases worldwide [65]. Understanding along with developing healthcare is essential for managing diabetes and its related problems, as many of the hypoglycemic agents currently used to treat have unwanted side effects [66]. Consequently, it is now crucial for scientific research to find new therapeutic options to address the symptoms of diabetes. It is commonly recognized that plant extracts can be used to treat diabetes, and a number of herbs have been established that work well in this area [67]. In this study, the blood glucose levels of the experimental animals were reduced in a significant manner by different fractions of the crude extract. The isolated compounds from this plant might be the causative factor behind this result. TNF-α, which is believed to cause insulin resistance and reduced insulin signal transduction, may be inhibited by phytol (**1**) and -amyrin (**4**) to combat diabetes [68,69]. Lupeol (**2**) might partially block α-glucosidase, α-amylase, and protein tyrosine phosphatase 1B (PTP 1B) activities to demonstrate anti-diabetic efficacy [69]. 

The biological properties of different fractions of *G. procumbens* crude MeOH extracts demonstrated the plant’s potential to be an important asset in terms of plant-based medicine. As was already noted, *G. procumbens* was found to be an abundant resource of secondary metabolites that show biologically activity. The isolated compounds should still be thoroughly studied to determine their safety profile and exact mode of action to elicit a pharmacological response. To extract more bioactive phytochemicals from this plant with therapeutic potential, extensive research is also needed.

## 3. Materials and Methods

### 3.1. Collection and Preparation of the Plant Material

The leaves of *G. procumbens* were collected from the Dhaka University campus, Bangladesh, in December 2016 and identified by an expert taxonomist of the Bangladesh National Herbarium, where a voucher specimen (DACB Accession No.: 45009) was deposited. The leaves of the plant were cleaned properly to remove dirt and other impurities. Then, it was shade-dried for several days and, utilizing a large-capacity grinding device, pulverized into a coarse powder.

### 3.2. Instrumentations, Drugs, and Chemicals

Sephadex LH 20 and Kieselgel 60H (Sigma-Aldrich, St. Louis, MI, USA) were used to carry out gel permeation chromatography (GPC) and vacuum liquid chromatography (VLC), respectively. Buchi Rotavapor (Essen, Germany) was used for evaporating solvent. Purification of the compounds was executed on precoated thin-layer chromatography (PTLC) plates (Silica gel 60 F 254, Darmstadt, Merck, Darmstadt, Germany). The spots on the thin-layer chromatography (TLC) plates were detected using UV light and vanillin/H_2_SO_4_ reagents. The NMR spectra were captured using a Bruker (400 MHz) instrument in deuterated chloroform (CDCl_3_). Every other substance utilized in the study, including solvents and reagents, was of the analytical variety and came from a reputable supplier (Merck, Germany; Active Fine Chemicals Ltd., Bangladesh; DaeJung, Korea). ASA, VS, Streptokinase (Popular Pharmaceuticals Ltd., Dhaka, Bangladesh), Glibenclamide (Diabenol, Square Pharmaceuticals Ltd., Dhaka, Bangladesh), Alloxan monohydrate (Sigma Chemical Co., St. Louis, MI, USA), normal saline solution (Beximco Infusion Ltd., Dhaka, Bangladesh), Tween-80 (BDH Chemical, London, UK), and an ACCU-ANSER DIGITAL Blood Glucose Meter were also used in the biological investigation.

### 3.3. Experimental Design

#### 3.3.1. Extraction of Plant Material

The powdered plant materials (950 gm) were extracted with distilled MeOH (3.0 L) for two weeks with random stirring and shaking. After two weeks, following a cotton filter, Whatman No. 1 filter paper was used to filter the entire mixture. The filtrate was thickened at 40°C with the help of a rotary evaporator. Finally, 29.0 gm of dried MeOH extract was obtained. Then, the EtOAc-soluble fraction and methanol-soluble fraction were separated from the crude methanol extract. 

#### 3.3.2. Isolation of Compounds

The concentrated EtOAc-soluble fraction (15.5 g) was exposed to VLC over silica gel using petroleum ether, dichloromethane, EtOAc, and MeOH with increasing polarity [70]. A total of 34 fractions of different polarities were eluted. Depending on the TLC plate behavior, VLC fractions 14, 15, and 16 (85% dichloromethane in petroleum ether, 100% dichloromethane, and 2% ethyl acetate in dichloromethane) were mixed together and other VLC fractions were obtained with 26% ethyl acetate in dichloromethane and 15% ethyl acetate in dichloromethane. These VLC fractions were further subjected to gel permeation chromatography (GPC) using a Sephadex (LH-20) column. A total of 16 sub-fractions were obtained. The sub-fractions 8–11 were mixed together and subjected to preparative thin-layer chromatography (PTLC) over silica gel (Kieselgel F254) using 1% ethyl acetate in toluene to yield phytol (**1**), lupeol (**2**), stigmasterol (**3**), and β-amyrin (**5**).

The VLC fraction 4 (2% dichloromethane in petroleum ether) was screened by TLC and this fraction showed white crystals. Then, the white crystals were collected by washing with n-hexane to yield fridelanol acetate (**4**). The VLC Fraction 17 (2% ethyl acetate in dichloromethane) showed needle-shaped crystals, and these crystals were purified by washing with n-hexane to yield a combination of stigmasterol and β-sitosterol (**6**).

#### 3.3.3. Preparation of Different Partitions for Biological Tests

Crude methanolic extract (CME) was fractionated using the modified Kupchan partitioning method [71] and the resultant partitionates were evaporated to dryness to yield petroleum-ether-soluble fraction (PESF), chloroform-soluble fraction (CSF), ethyl-acetate-soluble fraction (EASF), aqueous-soluble fraction (AQSF), and crude methanolic extract (CME) in a Rotary evaporator.

#### 3.3.4. Structural Identification of the Compounds

The ^1^H NMR and ^13^C NMR spectra of Compounds **1**–**6** in deuterated chloroform (CDCl_3_) were measured by the Bruker 400 NMR spectrometer at 400 MHz, and the values were explained in relation to the remaining non-deuterated solvent signal. The unit of measurement for coupling constants was Hertz (Hz). Chemical shifts were quantified in ppm.

#### 3.3.5. Test Animal Model

The experiment was conducted using 45 Wistar albino rats (110 ± 25 g), of either sex, aged 2–3 months, procured from Jahangirnagar University in Dhaka, Bangladesh. The rats were kept in standard environmental conditions in the Institute of Nutrition and Food Science (INFS) of the University of Dhaka and fed with a standard pellet diet and water ad libitum. Every day, the bowls of water and cages were cleaned and replenished. Before the test, the rats were allowed to stay in the experimental settings for a week to help them adjust. The experiment was conducted in accordance with the FELSA (Federation of European Laboratory Animal Science Associations) criteria. All of the studies were carried out in accordance with the institutional ethical committee’s approved procedures for the maintenance and utilization of laboratory animals [72]. The Ethical Review Committee of the Faculty of Pharmacy, University of Dhaka, Dhaka-1000, Bangladesh, issued its consent under the approval number Fa.Ph.E/002-A/22 prior to performing animal investigations. The “3R” (Replace, Reduce, and Refine) was strictly followed during the research and experiment design, and to avoid undue suffering, the experiment was carried out by skilled and experienced researchers and laboratory assistants. The mice models received an intraperitoneal overdose of ketamine HCl (100 mg/kg) and xylazine (7.5 mg/kg) at the conclusion of the experiment, followed by euthanasia [73].

#### 3.3.6. Acute Toxicity Study

The “Organization for Environmental Control Development” (OECD: Guidelines 420) fixed-dose method was used to conduct the oral acute toxicity test in laboratories under standard operating procedures [73]. Following the administration of high oral doses (2000 mg/kg) to the mice, a variety of parameters were recorded over the course of 72 h. The animals were watched closely for the first 30 min after treatment, with extra care used for the first 4 h, and then occasionally for the following 24 h to check for any harmful effects. The animals were seen and monitored for any changes in behavior, food intake, water intake, urination, respiration, body weight, convulsions, constipation, temperature change, changes in eye and skin colors, and animal death throughout the full observation period. Therefore, following oral administration of the *G. procumbens* crude methanolic extract (CME) and its different fractions (PESF, CSF, EASF, and AQSF), there was no death, behavioral alteration (sedation, excitability), allergic reaction, or any other major change. The dosage of 250 mg/kg b.w./day was selected for the antidiabetic activity investigation based on the safe dose adjustment from the perspective of oral acute toxicity.

### 3.4. Antioxidant Assay

#### DPPH Free Radical Scavenging Assay

To examine whether the plant extracts might scavenge free radicals from 1,1-diphenyl-2-picrylhydrazyl (DPPH), a total of 2.0 mL of a plant extract solution at various concentrations (ranging from 500 g/mL to 0.977 g/mL) was combined with 3.0 mL of a DPPH methanol solution (20 g/mL). The antioxidant activities of the plant extract were evaluated by comparing its ability to decolorize a purple-colored DPPH methanol solution to that of ascorbic acid (ASA) [72,73,74,75,76,77].

$$\%Inhibition\;of\;free\;radical\;DPPH=\left(1-\frac{Absorbance\;of\;sample}{Absorbance\;of\;the\;control\;reaction}\right)\times 100$$

### 3.5. Cytotoxicity Assay

#### Brine Shrimp Lethality Bioassay

The brine shrimp lethality assay was used to evaluate the prospective cytotoxicity of different extracts from plants. A total of 38 g of NaCl salt and 1000 mL of distilled water were combined with NaOH to keep the pH constant (8.0) in order to replicate seawater. Eggs of brine shrimp were nurtured to become nauplii in the synthetic saltwater. Dimethyl sulfoxide (DMSO) was reduced to various quantities (400 g/mL to 0.78125 g/mL) before being added to the test samples. With DMSO serving as the negative control, vincristine sulfate was employed as the standard for comparison in a range of doses (400 g/mL to 0.78125 g/mL). After counting the nauplii visually, 5 mL of simulated seawater was added to the mixture in vials [72,74,76,77].

(1) $$Mortality\left(\%\right)=\frac{Number\;of\;nauplii\;death}{Number\;of\;nauplii\;taken}\times 100$$

### 3.6. In Vitro Thrombolytic Assay

This experiment was carried out adopting the methodology described by Ahmed et al. [78]. Venous blood samples of 10 mL were collected from healthy individuals. A sterile Eppendorf tube containing preweighed blood was filled with blood at a rate of 0.5 mL per tube. After 45 min of incubating at 37 °C, coagulation began. Except for the clot, all of the generated serum was removed. Each tube was weighed once again to determine the clot weight.

Clot weight = weight of clot containing tube − weight of tube alone

In total, 100 μL of solution of extracts were collected in the tubes separately. Then, as a positive control, 100 L of streptokinase had been incorporated simultaneously. Next, 100 mL of distilled water was introduced to the group of empty tubes separately. Each tube was incubated at 37 °C for 90 min. The difference between the weights taken prior to and following clot lysis was represented as a percentage, as shown below.

% clot lysis = (Weight of the clot after lysis/Weight of clot before lysis) × 100

### 3.7. Antidiabetic Assay

#### Alloxan-Induced Diabetic Test

The anti-diabetic activity was investigated in alloxan-induced diabetic rats. Here, Glibenclamide acted as a reference standard (positive control).

Prior to injection, alloxan monohydrate was first individually weighed for each animal in accordance with their weight and then dissolved with 0.2 mL or 200 L saline. By intraperitoneally administering it at a dose of 120–150 mg/kg body weight, diabetes developed. After 72 h of observation, blood glucose levels were tested in the animals employing an ACCU-ANSER DIGITAL Blood Glucose Meter. Out of forty-five rats, three rats did not respond to the alloxan treatment, and two rats died. Finally, 40 rats were divided into eight groups for experimental study, with each group containing 5 rats. Diabetic rats were defined as those with blood glucose levels exceeding 162 mg/dL (9.0 mmol/L), and they were employed in the investigation.

The rats in Group I were in good health and acted as the non-diabetic group serving as the control. Rats that had developed diabetes after being exposed to alloxan made up Groups II to Group VIII. Group II acted as a diabetic, untreated, or adverse control group. Animals in Group III, which acted as the diabetic positive control group, received oral administration of the reference medication glibenclamide (Diabenol, Square Pharmaceuticals Ltd., 5 mg/kg b.w./day). The crude methanolic extract (CME) of *G. procumbens* and its various fractions (petroleum ether, chloroform, ethyl acetate, and aqueous soluble fraction), each at a dose of 250 mg/kg b.w./day, were force-fed orally to the rats in Groups IV to VIII. Three weeks of prescribed treatments (1 mL/rat) were given to the rats [79]. Before being administered orally to the test animals, crude methanolic extract and its various components, as well as glibenclamide, were suspended in distilled water. Only the vehicle (distilled water) was administered to Groups I and II. On day 0 (the beginning of treatment), day 7, day 14, and day 24 (the final day of treatment), fasting blood glucose levels were measured. On days 0, 7, 14, and 21, blood samples from the overnight starved rats were taken from their tail veins to measure their blood glucose levels using a glucometer.

## 4. Conclusions

The methanolic extract of the leaves of *G. procumbens* was investigated for the isolation of the potent secondary metabolites of this plant. Six compounds were produced after several steps of chromatographic separation as well as purification: phytol (**1**), lupeol (**2**), stigmasterol (**3**), friedelanol acetate (**4**), β-amyrin (**5**), and a mixture of stigmasterol and β-sitosterol (**6**). For the biological investigation, the crude methanol extract was first fractionated by partitioning it into petroleum ether (PESF)-, chloroform (CSF)-, ethyl acetate (EASF)-, and aqueous-soluble fractions (AQSF), and various biological assays, such as cytotoxicity, anti-diabetic, thrombolytic, antioxidant, and antimicrobial activity, were carried out. In the DPPH free radical scavenging assay, the EASF of the *G. procumbens* leaves demonstrated substantial free radical scavenging activity, with an IC_50_ value of 10.78 g/mL compared to the standard ascorbic acid (ASA) with an IC_50_ value of 31.25 g/mL. In the brine shrimp lethality bioassay, the CSF exhibited the highest cytotoxic effect with an LC_50_ value of 1.94 µg/mL, compared to the standard vincristine sulphate (VS) with an LC_50_ value of 0.464 µg/mL. In the assay for thrombolytic activity, the crude methanolic extract demonstrated significant clot lysis (63.77%), whereas the standard streptokinase showed 70.78%. The PESF revealed the highest glucose-lowering activity of 70.37% (5.78 mmol/L), compared to the standard glibenclamide, which showed 63.24% (5.16 mmol/L). The results of the bioassays make it abundantly obvious that this plant’s leaves have substantial antioxidant, cytotoxic, thrombolytic, and anti-diabetic effects. Therefore, taking into account its prospective medicinal properties, the plant can be extensively examined and assessed in order to determine its inherent efficacy and substantiate its adoption as medicine.

## Figures and Tables

**Table 1:** Free radical scavenging, cytotoxicity, and thrombolytic activities of *G. procumbens*.

	Sample/ Standard	DPPH Free Radical Scavenging Activity (IC_50_ µg/mL)	Cytotoxicity (LC_50_ µg/mL)	% Clot Lysis
Extract	CME	25.68 ± 0.10	3.94 ± 0.16	63.77 ± 0.23
	PESF	113.89 ± 3.25	3.45 ± 0.40	48.62 ± 0.56
	CSF	85.55 ± 2.74	1.94 ± 0.24	44.10 ± 0.40
	EASF	10.78 ± 0.11	5.59 ± 0.70	48.17 ± 0.58
	AQSF	93.09 ± 2.19	2.07 ± 0.36	26.00 ± 0.74
Standard	Ascorbic acid	31.25 ± 0.67	-	-
	VS	-	0.464 ±0.02	-
	Blank	-	-	2.00 ± 0.16
	SK	-	-	70.78 ±0.46

Values are expressed as mean ± SD (n = 3). CME = Crude methanolic extract, PESF = Petroleum-ether soluble fraction, CSF = Chloroform soluble fraction, EASF = Ethyl acetate soluble fraction, AQSF = Aqueous soluble fraction, VS = Vincristine sulphate, SK = Streptokinase (positive control), Water (negative control for thrombolytic activity).

**Table 2:** Effects of *G. procumbens* crude methanolic extract and its different fractions on fasting blood glucose levels in alloxan-induced diabetic rats.

Test Models	Groups	Fasting Blood Glucose Levels (mmol/L) Mean± SEM			
		Day 0	Day 7	Day 14	Day 21
Normal	Normal Control	5.66 ± 0.2379	5.74 ± 0.2421	5.58 ± 0.1772	5.68 ± 0.3308
Diabetic	Diabetic Control	16.56 ± 2.164	20.06 ± 2.355	21.54 ± 2.808	22.52 ± 2.161
	Glibenclamide	14.24 ± 2.106	5.66 ± 0.3906	4.88 ± 0.3734	5.16 ± 0.3669
	CME	13.70 ± 1.204	5.86 ± 0.2159	7.38 ± 0.3007	6.12 ± 0.2131
	PESF	23.72 ± 3.031	7.18 ± 0.4259	5.98 ± 0.2818	5.78 ± 0.2956
	CSF	13.72 ± 2.213	6.02 ± 0.3137	4.24 ± 0.2768	5.08 ± 0.2059
	EASF	17.6 ± 2.385	11.72 ± 2.835	6.44 ± 0.5819	6.2 ± 0.6050
	AQSF	19.74 ± 1.076	9.8 ± 2.852	7.66 ± 2.040	5.6 ± 0.2665

Values are given as mean ± S.E.M. Diabetic controls were compared with normal controls and treated groups at a corresponding time interval.

**Table 3:** % Reduction in blood glucose level by test materials.

% Reduction in Blood Glucose Level (BGL)				
Sample/Standard	After 7 Day	After 14 Day	After 21 Day	% Reduction in BGL (Average)
Glibenclamide (STD)	60.25	65.73	63.76	63.24
CME	57.22	45.93	55.32	52.82
PESF	69.73	67.20	74.20	70.37
CSF	56.12	69.09	62.97	62.72
EASF	33.40	63.40	64.77	53.85
AQSF	50.35	60.99	71.63	60.99

CME = Crude methanolic extract, PESF = Petroleum-ether-soluble fraction, CSF = Chloroform-soluble fraction, EASF = Ethyl-acetate-soluble fraction, AQSF = Aqueous-soluble fraction.

## Data Availability

Not applicable.

## Supplementary Materials

The following supporting information can be downloaded at: https://www.mdpi.com/article/10.3390/molecules28104186/s1, Figure S1: ^1^H NMR spectrum (400 MHz, CDCl_3_) of Compound 1 (Phytol); Figure S2: Partially Expanded ^1^H NMR spectrum (400 MHz, CDCl_3_) of Compound **1** (Phytol); Figure S3: ^13^C NMR spectrum (100 MHz, CDCl_3_) of Compound **1** (Phytol); Figure S4: Partially Expanded ^1^H NMR spectrum (400 MHz, CDCl_3_) of Compound **2** (Lupeol); Figure S5: Partially Expanded ^1^H NMR spectrum (400 MHz, CDCl_3_) of Compound **3** (Stigmasterol); Figure S6: Partially Expanded ^1^H NMR spectrum (400 MHz, CDCl_3_) of Compound **4** (Friedelanol Acetate); Figure S7: Partially Expanded ^1^H NMR spectrum (400 MHz, CDCl_3_) of Compound **5** (β-Amyrin); Figure S8: Partially Expanded ^1^H NMR spectrum (400 MHz, CDCl_3_) of Compound **6** (β-Sitosterol).

## Sample Availability

Samples of the compounds are available from the authors.
