# Supplementary material for: Phytochemical and Biological Investigation of an Indigenous Plant of Bangladesh, Gynura procumbens (Lour.) Merr.: Drug Discovery from Nature
Source: Molecules. 2023 May 19;28(10):4186. doi: 10.3390/molecules28104186 (PMC10221986; doi:10.3390/molecules28104186)
Supplement: Supplementary file 1 [file molecules-28-04186-s001.zip › molecules-2268480-supplementary.pptx]

## Slide 1
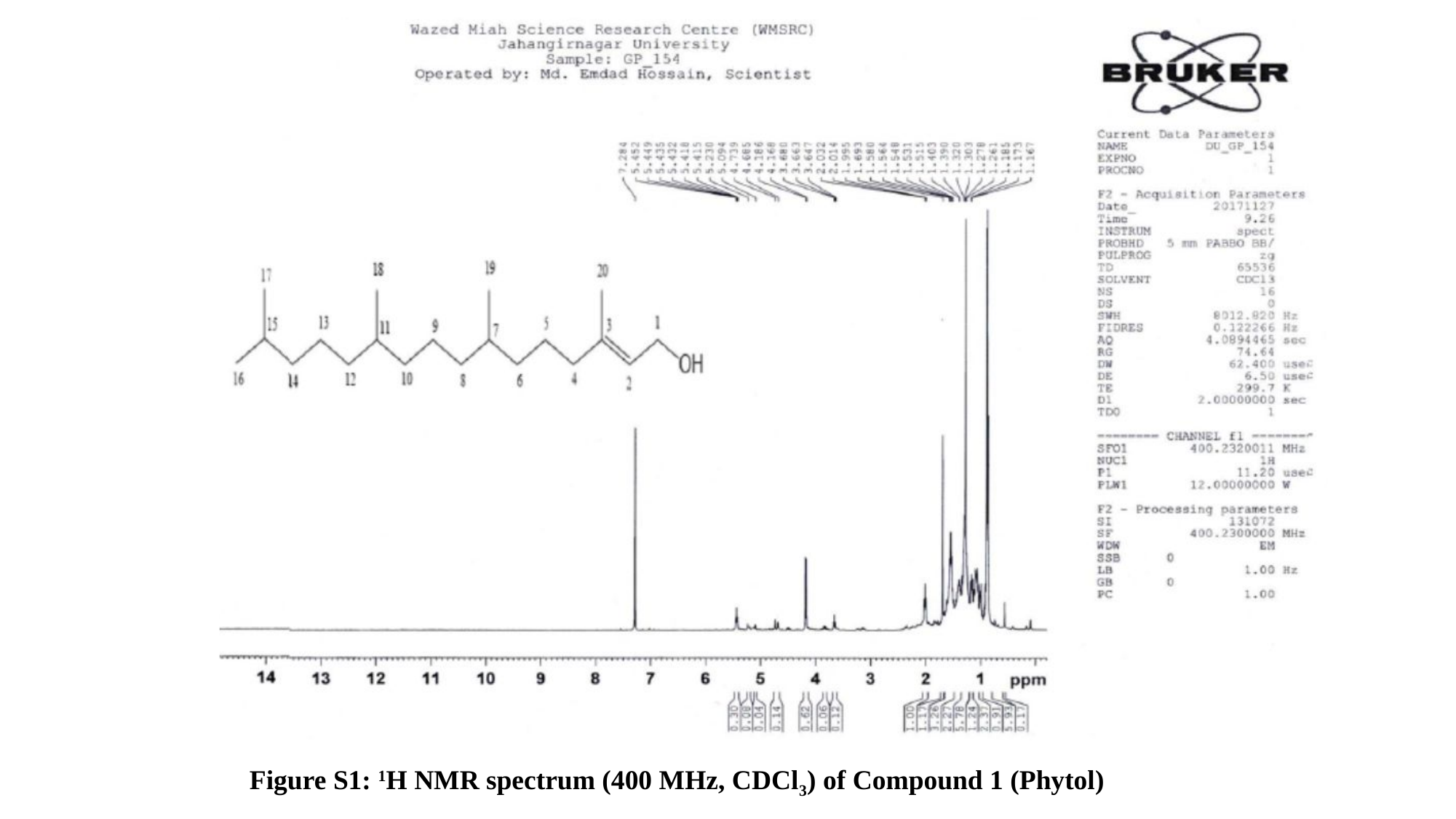

Figure S1: 1H NMR spectrum (400 MHz, CDCl3) of Compound 1 (Phytol)

## Slide 2
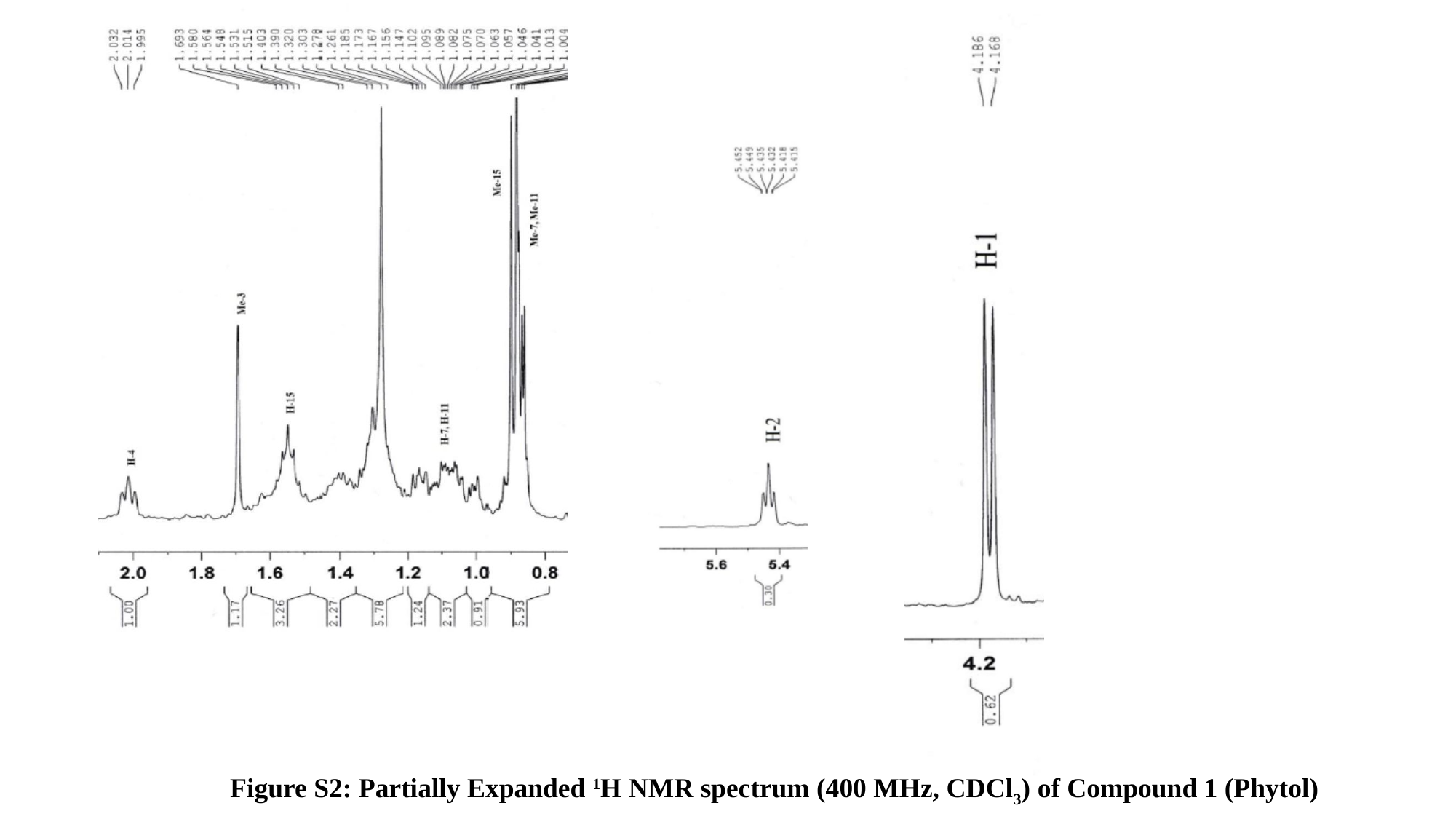

Figure S2: Partially Expanded 1H NMR spectrum (400 MHz, CDCl3) of Compound 1 (Phytol)

## Slide 3
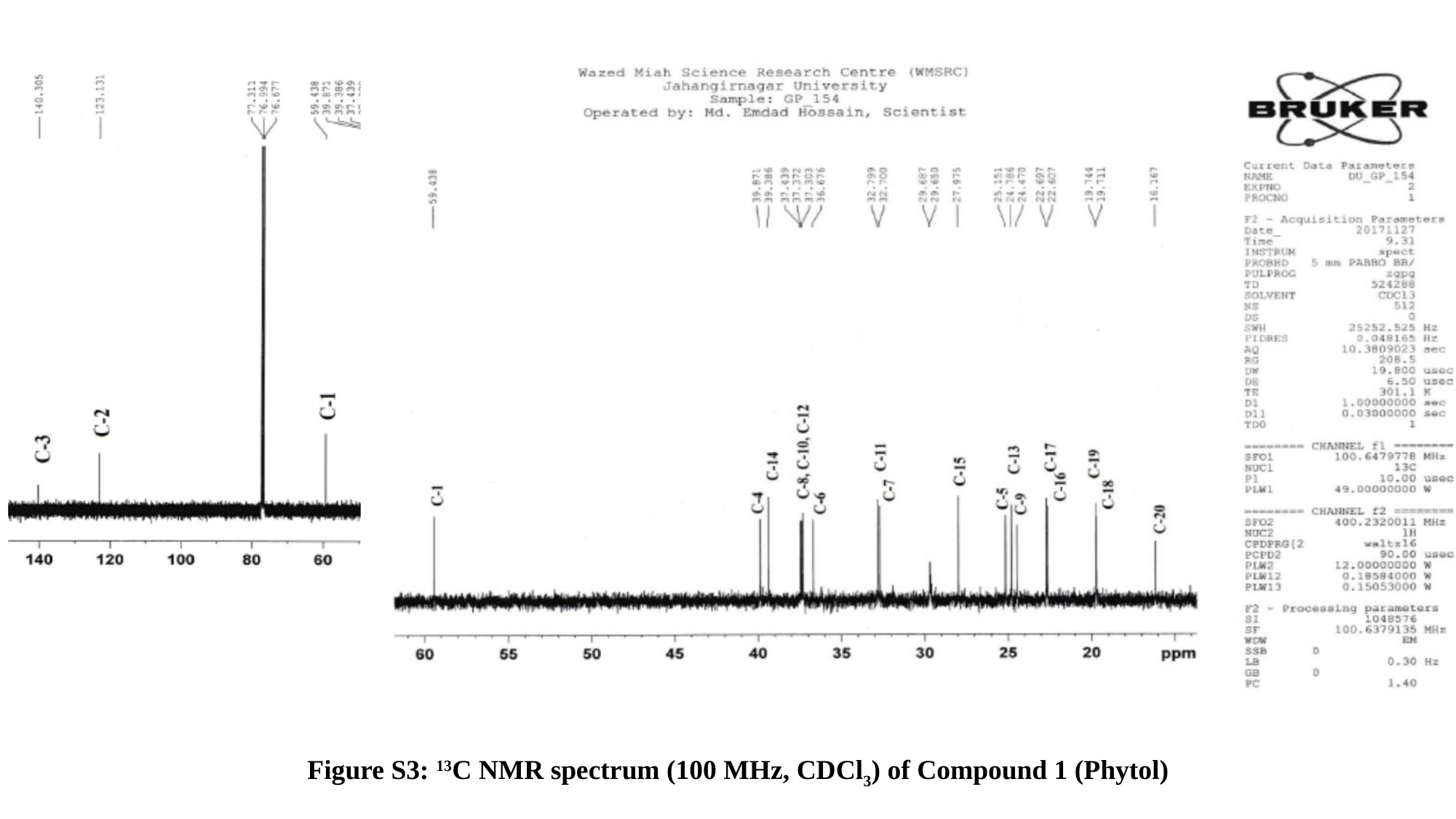

Figure S3: 13C NMR spectrum (100 MHz, CDCl3) of Compound 1 (Phytol)

## Slide 4
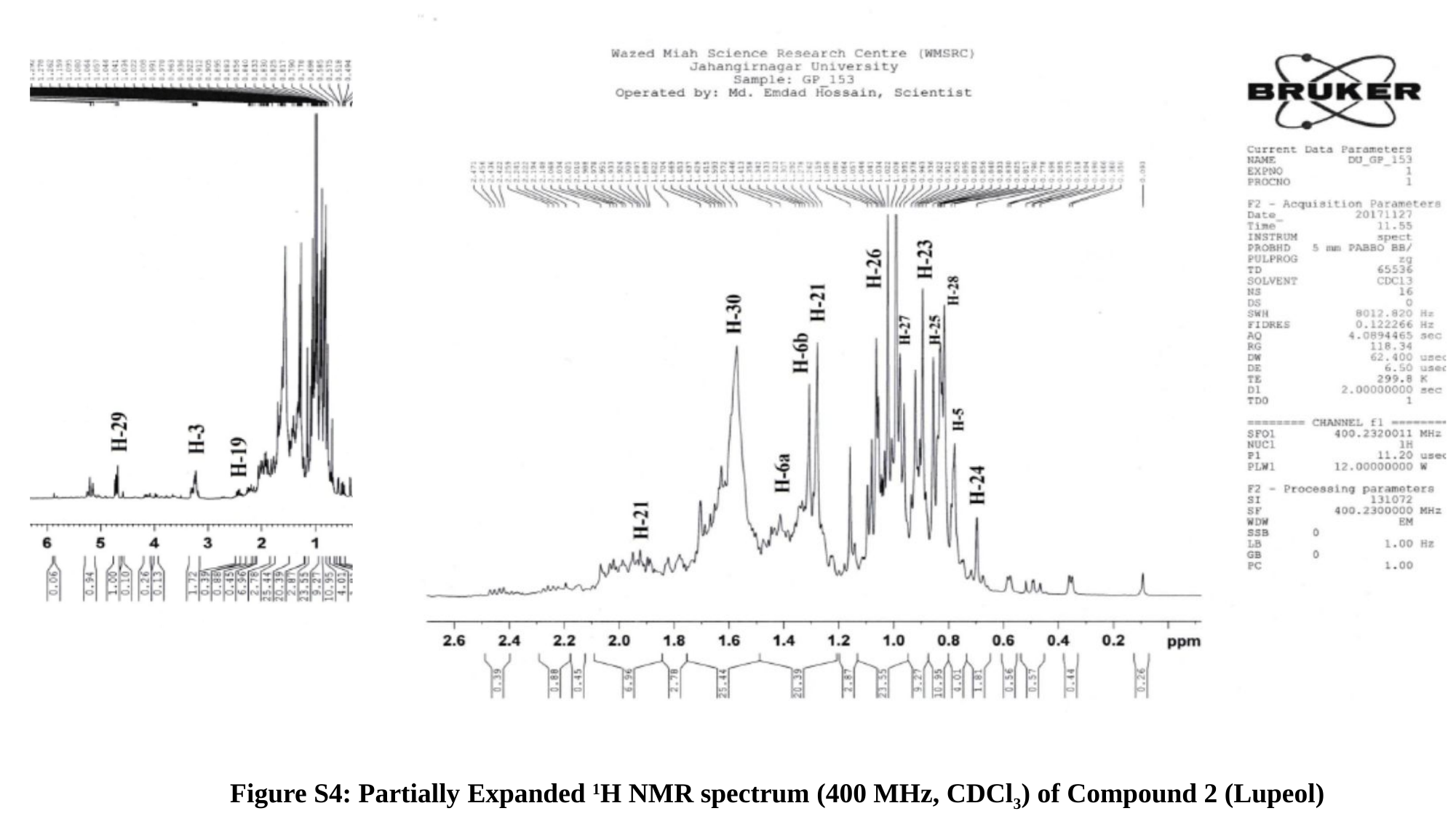

Figure S4: Partially Expanded 1H NMR spectrum (400 MHz, CDCl3) of Compound 2 (Lupeol)

## Slide 5
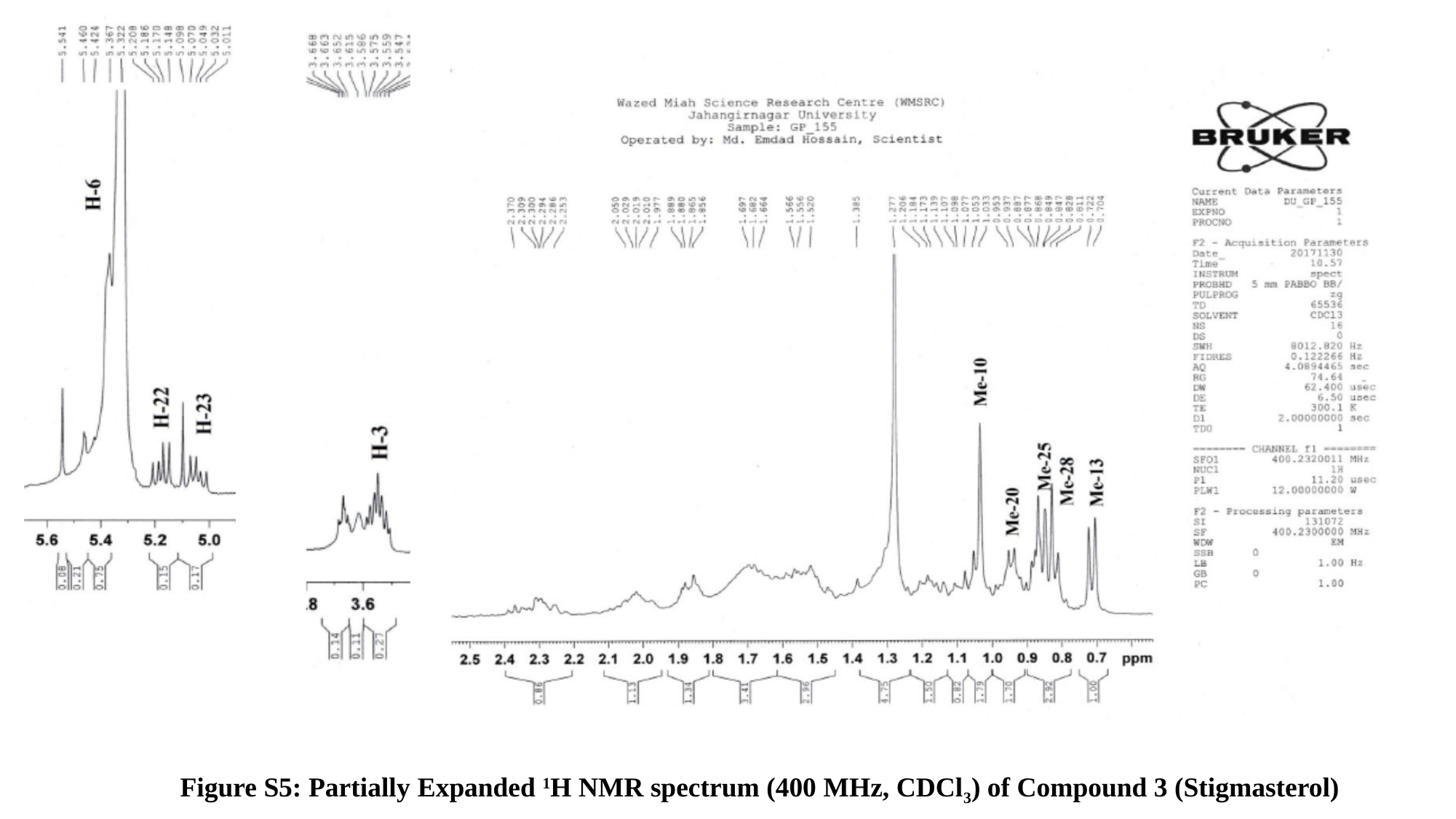

Figure S5: Partially Expanded 1H NMR spectrum (400 MHz, CDCl3) of Compound 3 (Stigmasterol)

## Slide 6
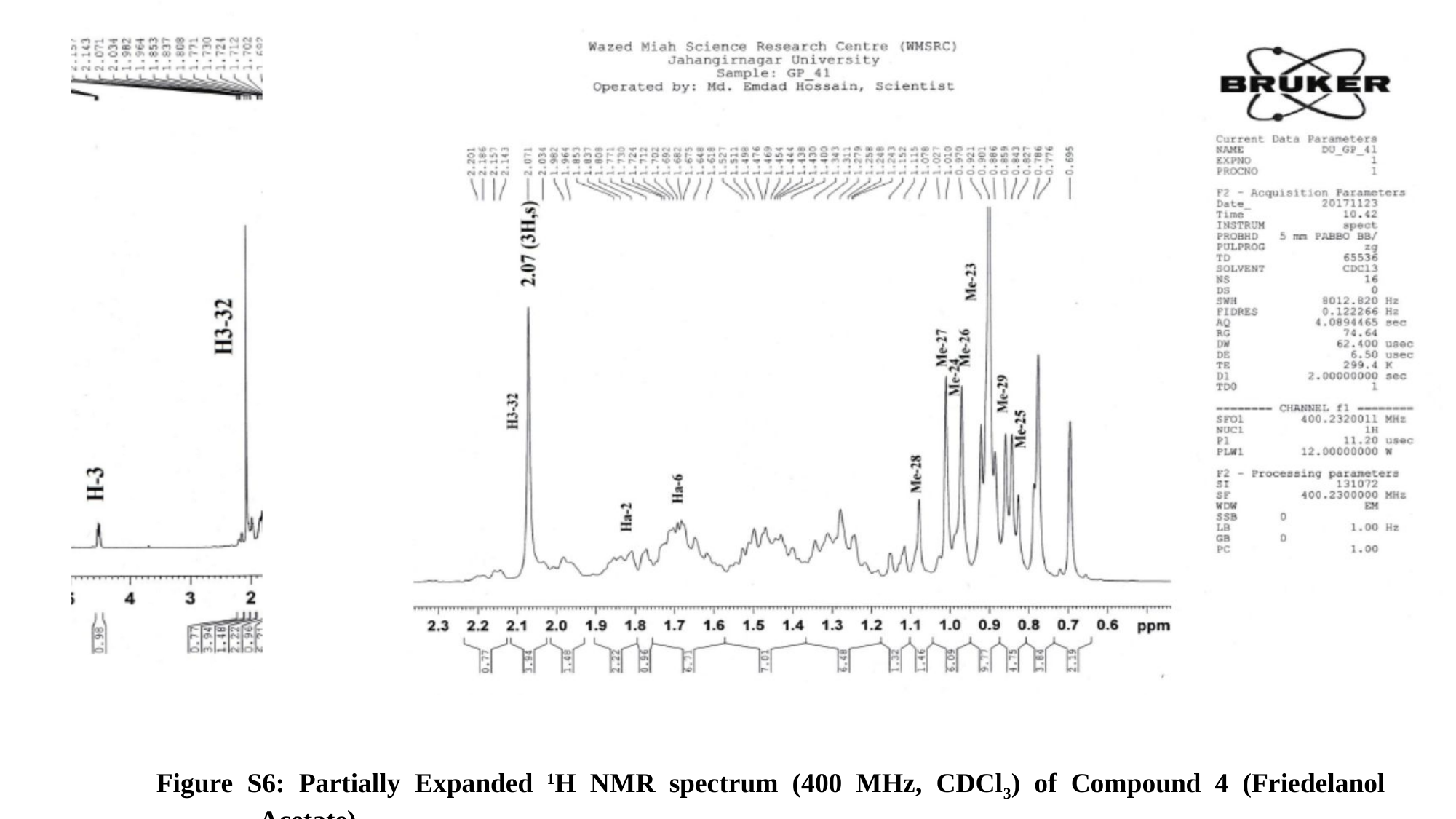

Figure S6: Partially Expanded 1H NMR spectrum (400 MHz, CDCl3) of Compound 4 (Friedelanol Acetate)

## Slide 7
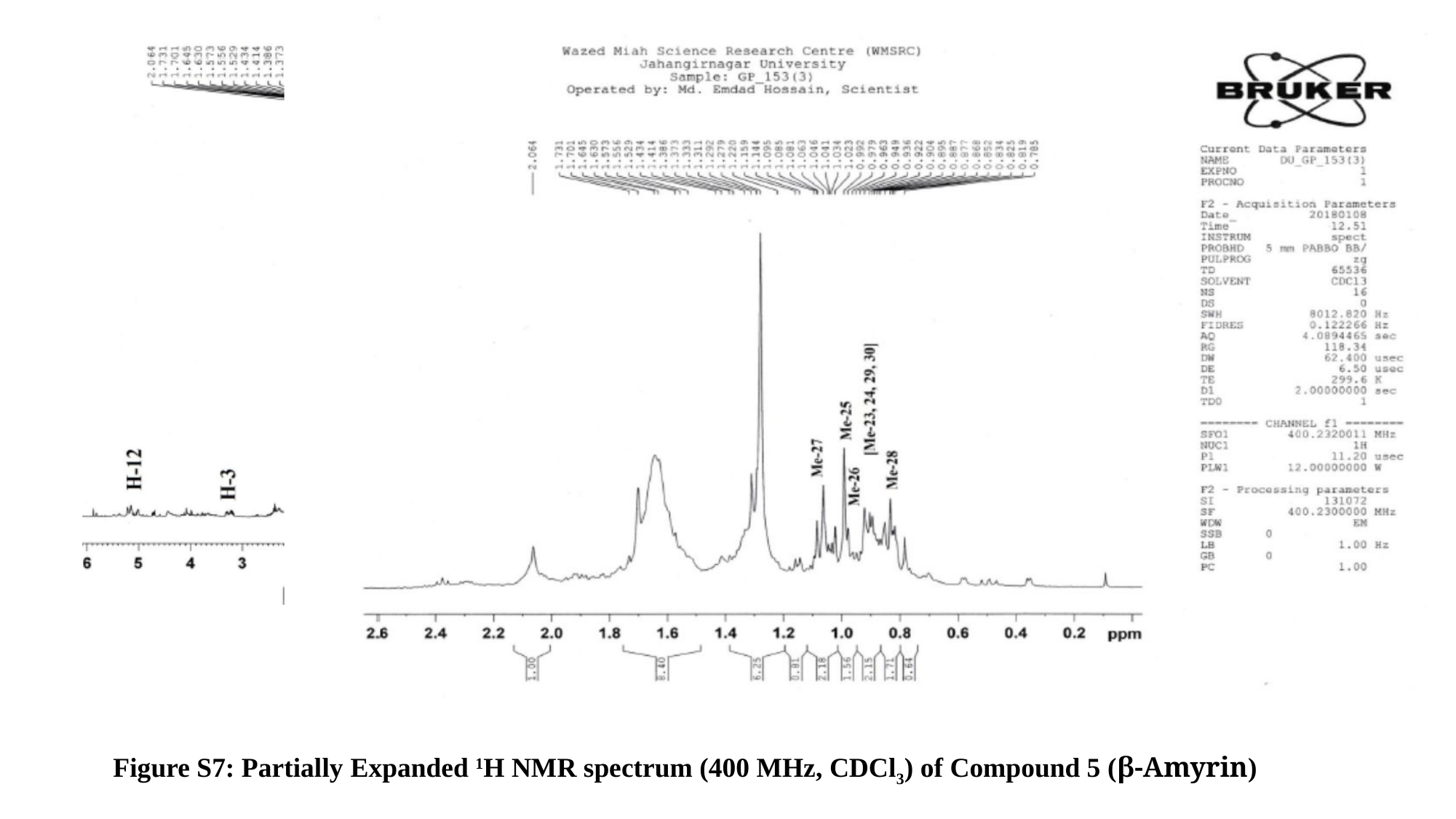

Figure S7: Partially Expanded 1H NMR spectrum (400 MHz, CDCl3) of Compound 5 (β-Amyrin)

## Slide 8
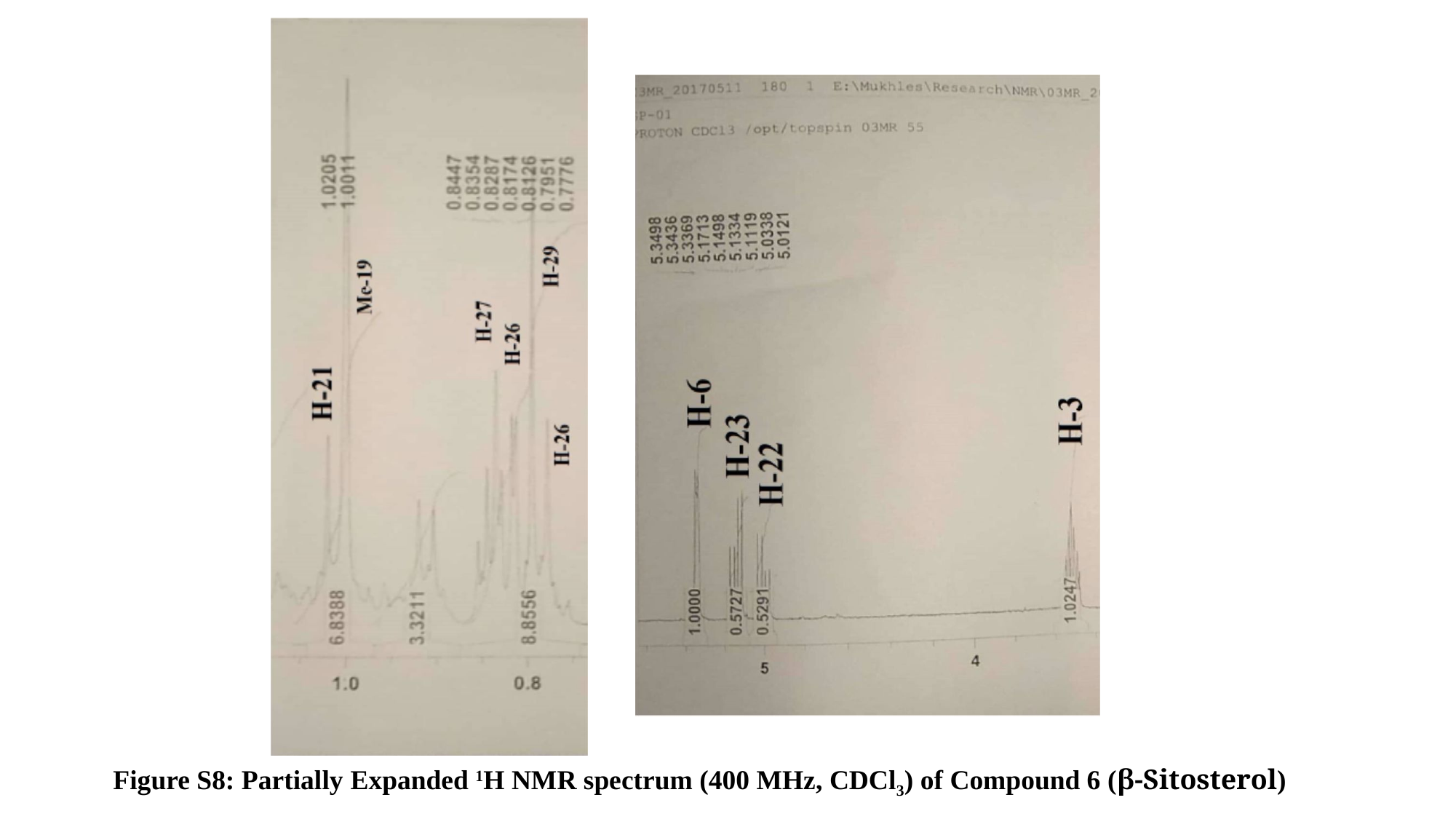

Figure S8: Partially Expanded 1H NMR spectrum (400 MHz, CDCl3) of Compound 6 (β-Sitosterol)
